# Continuous Quality Improvement in Daily Clinical Practice: A Proof of Concept Study

**DOI:** 10.1371/journal.pone.0097066

**Published:** 2014-05-20

**Authors:** Jonathan A. Lorch, Victor E. Pollak

**Affiliations:** 1 Department of Medicine, Weill Cornell Medical College and The Rogosin Institute, New York, New York, United States of America; 2 Department of Medicine, University of Colorado Health Sciences Center, Aurora, Colorado, United States of America; 3 MIQS, Inc., Boulder, Colorado, United States of America; University of Colorado, United States of America

## Abstract

Continuous Quality Improvement (CQI) is an iterative process of: planning to improve a product or process, plan implementation, analyzing and comparing results against those expected, and corrective action on differences between actual and expected results. It is little used in clinical medicine. Anemia, a complex problem in End Stage Renal Disease patients, served to test the ability of an unique electronic medical record (EMR) optimized for daily care to empower CQI in practice. We used data collected during daily care, stored in the EMR, and organized to display temporal relationships between clinical, laboratory, and therapeutic events. Our aims were optimal hemoglobin with minimum epoetin, and maintaining stable hemoglobin and epoetin. The study was done on 250 patients treated by maintenance hemodialysis (HD), receiving epoetin prior to February 1, 2010 and followed to July 31, 2011. Repleting iron, ensuring iron sufficiency, slow epoetin reduction, and decision support tools enabling data display over long periods in patient-centered reports were key elements. Epoetin dose, adjusted 6–8 weekly, was based on current clinical conditions and past responses. Hemoglobin increased by months 1–2; epoetin decreased from month 4. By months 16–18, epoetin had decreased 42% to 9,720 units/week while hemoglobin increased 8% to 123.6 g/L. Hemoglobin and epoetin were stable from month 7 onward. New epoetin orders decreased 83%. Transferrin saturation increased after the study start. Individual patient hemoglobin variation decreased by 23%, range by 27%. Mortality, 11.78 per 100 patient years, was 42% less than United States dialysis patient mortality. Allowable epoetin charges decreased by $15.33 per treatment and were $22.88 less than current Medicare allowance. The study validates the hypothesis that an EMR optimized for daily patient care can empower CQI in clinical medicine and serve to monitor medical care quality and cost.

## Introduction

The statistical approach to continuous quality improvement (CQI) was invented by Shewhart over 50 years ago [1], and applied in industrial manufacturing and service industries by W. Edwards Deming [2]. He demonstrated repeatedly its capacity to improve quality and reduce costs of manufacturing and services. CQI is an iterative and continuing process of improvement that involves: (1) planning to improve a product or process, (2) implementation of the plan, (3) analyzing results and comparing results against those expected, and (4) taking corrective action on significant differences between actual and expected results. Critical to effective CQI implementation are, among others: deciding to adopt the CQI philosophy, constancy of purpose toward improvement of service, leadership to help people do a better job, training to achieve that end, and an education and self-improvement program [2]. At its heart, CQI concerns itself with taming variability. In industry the focus is on processes that affect quality of the final product, whereas in clinical medicine it is on the varied responses to therapeutic interventions determined at least in part by the biology of individual patients, their co-morbidities, and details of their illnesses.

Care of chronic diseases that now predominate in medical practice accounts for >75% of US medical care costs [3]. In chronic disease, Fries wrote over 35 years ago “a major failure of the traditional chart is its inability to indicate adequately complex temporal relationships between clinical, laboratory, and therapeutic events” [4]. One of us (VEP) postulated that “successful management and treatment of patients and the important individual manifestations of a chronic disease require complex feedback systems that relate therapeutic interventions to clinical and laboratory information relevant to multi-organ systems over prolonged periods” [5]. Although testing this postulate seems to us to depend at least in part on collecting and viewing relevant data sequentially over time, this process is far from routine. We found little literature on CQI in clinical medicine, and sparse reports on the effect of electronic medical records (EMRs) on quality improvement in any chronic disease [6].

The patient-centered, comprehensive, coded, analyzable EMR used herein, first developed 30 years ago, was designed specifically to enable CQI in medical practice [5], [7]. Discrete practice details for diseases affecting multiple organ systems were transformed into coded data, enabling rearrangement of data elements at the point-of-care to facilitate clinical practice and observations over many years.

End Stage Renal Disease (ESRD) is complex, costly, and affects >600,000 patients in the United States [8]. Because ESRD is highly regulated with mandated criteria to start dialysis treatment and frequent State and Federal oversight, ESRD has unique advantages as a model for a proof of concept study. Treatments, devices, drugs used, and dialysis procedures are largely standardized, but outcomes vary greatly between dialysis facilities [9] even though standardized treatment protocols are used widely.

In protocol-driven health care delivery some individuals will not benefit from the parameters of the protocol; without individual scrutiny of care choices patients could actually be harmed. Increasing epoetin alfa (EPO) given to HD patients has led to administration of very large amounts, now acknowledged to adversely affect survival at least in patients with moderate anemia (Hb 100–130 g/L) [10], [11].

For the present study we examine the effects of treatment of anemia, a chronic condition in HD patients with ESRD. Underlying causes include deficiency of iron stores and the erythrocyte stimulating hormone erythropoetin. EPO has played a major role in anemia treatment for >20 years [12]. Adequate iron stores are needed for optimal EPO response [13]. Iron deficiency is common in patients treated by HD [14] and those with decreased renal function not on dialysis [15]. Yet many studies of EPO use in chronic renal failure were done on patients iron deficient when EPO was administered [16]–[18]. When EPO is administered to patients deficient or marginally sufficient in iron, demands for iron for hemoglobin (Hb) production are likely to deplete iron available for other essential metabolic processes.

We have reported favorable effects of treatment with IV iron but not with oral iron [14]. Later, retrospective analysis of clinical practice from 1998 to 2007 clearly demonstrated the importance of iron in long-term survival of HD patients, and that survival was better with relatively low EPO doses [19]. The practice at that time was to administer EPO using a standardized protocol in doses aimed to maintain patient Hb within the 100–120 g/L range; to decrease or withhold EPO solely on when the latest patient Hb was >125 or >130 g/L; and to resume and even increase EPO administration when Hb had not responded or when the latest Hb decreased to <90 or <100 g/L [20].

Analysis showed that this approach was associated with wide Hb swings and frequent prolonged periods with low Hb levels. We therefore developed a prospective patient-centered CQI approach, and report on its design and testing under the leadership of one of the authors (JAL). We made use of patient-specific data collected during daily care and stored in the EMR. Our objectives were: to achieve optimal Hb with minimum required EPO, and maintain stable Hb and EPO dose.

## Methods

### The Participants

The study was done on all 264 patients treated by maintenance in-center HD in one of three dialysis unit managed by The Rogosin Institute (New York, NY) and affiliated with New York Presbyterian Hospital and Weill Medical College of Cornell University. Before February 2010, intravenous (IV) iron and EPO had been prescribed using a protocol consistent with the Kidney Disease Outcomes Quality Initiative (KDOQI) guidelines [20], and influenced by Centers for Medicare and Medicaid Services (CMS) mandates. The 250 patients, who had been receiving EPO in October 2009 to February 2010, are the subjects of this analysis. They were followed until July 31, 2011.

### The EMR

**Figure 1:**
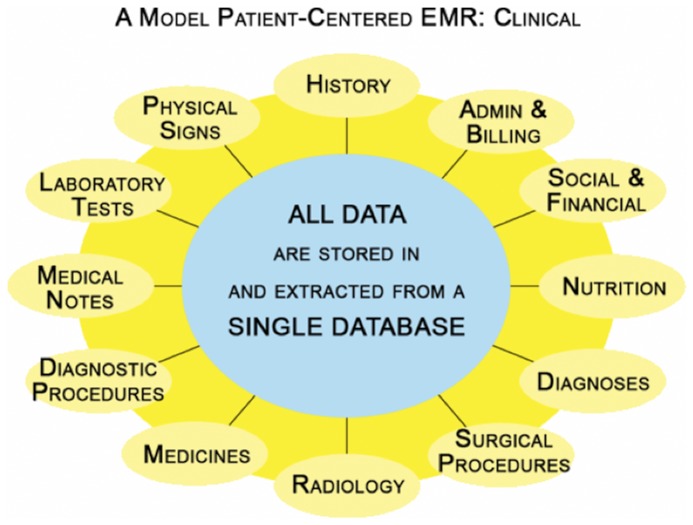
A model of a patient-centered EMR that, with a single database, accommodates multiple clinical functions and domains of information. As shown these include symptoms, physical signs, laboratory tests, medicines, diagnoses, diagnostic and surgical procedures, radiology, medical notes, nutrition, and social work. The single database also accommodates and includes the needs of: caregivers and other staff; data entry and reporting of physicians, nurses, dietitians, social workers, pharmacists, administrators, and clerical, billing, and accounting staff; multiple locations including main and remote dialysis units, peritoneal dialysis unit, home HD, chronic kidney disease, renal and other transplant, physician office, hospital outpatient; non-clinical functions such as insurance, fee tables, charges, physician billing, dialysis unit billing, electronic remittance and explanation of benefits.

The EMR (MIQS Inc, Boulder, CO) employs a relational database (SAP Sybase Adaptive Server Enterprise, SAP AG, Walldorf, Germany), running on a server computer (Oracle, Redwood Shores CA). Its architecture is shown diagrammatically in Figure 1. It is accessed using a custom toolset (4D, Inc., San Jose, CA) from client personal computers in dialysis units and other practice sites [21]. All clinical, administrative, and financial data are immediately accessible at all times on patients ever entered into the database. Subject to security considerations, lifetime patient data relevant to pertinent caregiver needs are accessed whenever and wherever needed.

Coded data elements include diagnoses, procedures, symptoms, signs, medications, allergies, and hospitalizations. HD treatment screens record all details at the time of treatment. HD orders and medications to be given during HD automatically populate treatment screen fields.

Accuracy and completeness of data on EPO and IV iron administered during HD treatments are facilitated and ensured by full software integration of billing and clinical information. Accuracy and completeness of data on hospital admissions are also facilitated and ensured by software integration of billing and clinical information.

To provide clinically useful point-of-care reports MIQS-designed embedded query tools are incorporated. They provide classes of reports (including single patient tabular, multi-patient tabular, and aggregate reports) to organize data quickly in any way desired over any time span, to make knowledge available about individual patients, and groups. User created reports can be updated and organized at the point-of-care, and are designed to facilitate clinical decisions based on timely, complete, relevant, patient-specific, time-oriented data. Individual patient reports include contemporaneous medications and laboratory tests, comprehensive lifetime lists of diagnoses, surgical procedures, diagnostic procedures, allergies, adverse drug reactions, immunizations, and hospitalizations.

### The Framework for the CQI Study

Based on our previous study [19] we developed a framework for hematinic medication administration in individual patients treated by HD. It was not a protocol to be applied uniformly to a heterogeneous patient population. Rather, the elements of the framework represented goals that had been shown to result in optimum patient survival using a minimum amount of EPO. To achieve the desired goals required knowledge of each patient, familiarity with factors causing apparent EPO resistance, and visual access in a single report to the domains of data that might effect application of the framework in individual patients.

The goals within the framework included: Iron replete patients were to receive IV iron 200 mg monthly as a single dose. Patients not iron sufficient (TSAT <25% and/or serum ferritin <300 µg/L) were to receive iron 200 mg during 5 consecutive HD treatments, thereafter 200 mg monthly. EPO and iron dose changes were to be evaluated monthly. EPO changes were to be made only 6–8 weekly as normal erythrocyte lifespan is ≈120 days [22], and because we had observed that large Hb variations accompanied frequent EPO changes.

Other details were:

- Patients with Hb<120 g/L and/or EPO>27,000 units/week were examined and data reviewed to determine possible cause(s) for EPO resistance. If considered resistant EPO dose was held unchanged until resistance judged by clinical examination, increased serum albumin and decreased C reactive protein, was considered unlikely.
- In patients unresponsive to EPO, the dose was reduced stepwise to the last level at which a response to EPO occurred.
- Before EPO was increased, a previously responsive patient with stable Hb<115 g/L was evaluated for inflammatory states and other possible reasons for unresponsiveness.
- If Hb was <110 g/L, EPO dose changes were made to try to attain a new goal of 120 g/L.
- If not iron sufficient, IV iron was given to achieve iron sufficiency, leaving EPO dose unchanged. EPO dose was changed only after iron sufficiency was established and maintenance iron initiated.
- If Hb was >125 g/L on 3 consecutive occasions EPO was reduced by 20%, but never to 0 (zero).
- EPO dose was not to be raised to >27,000 units/week without close consideration.
- When iron sufficient, EPO naive patients received EPO 90 units/Kg/week initially. The dose was not adjusted for 6–8 weeks, and the starting dose was not changed before there was evidence of iron sufficiency. Once a response was achieved (Hb 115–125 g/L), EPO was reduced by 20%. With assurance of iron repletion and sufficiency, EPO dose was then modified as in EPO established patients.

### Implementation

**Figure 2:**
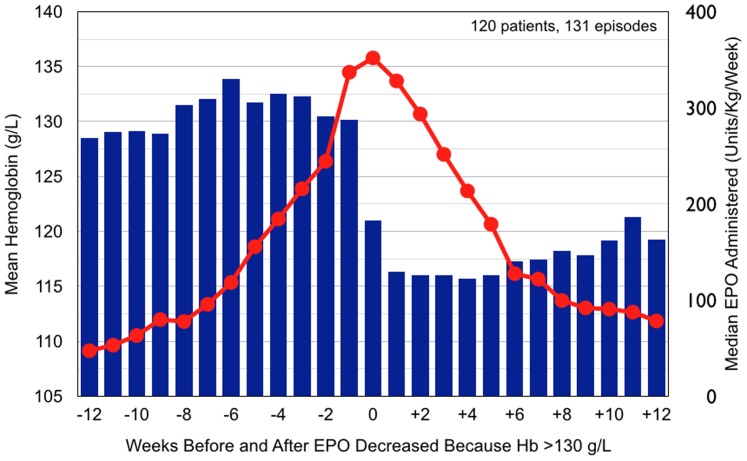
Summary of findings for 12 weeks before and after EPO administration was decreased because Hb was >130 g/L in 120 patients (131 episodes) in the study dialysis unit during the protocol period in calendar year 2009. EPO reduction (blue bars) was followed by pronounced and prolonged reduction of Hb (red dots) to <115 g/L. Similar observations were made in 2 other dialysis units that deployed the EMR.

Implementation required a new approach to prescribing hematinic drugs and managing the process. The prevailing practice had been that the medical director or attending physicians signed EPO orders previously written by designated nurses. Guiding the protocol used for EPO prescription was the then current CMS policy that penalized reimbursement when the Hb was >130 g/L. The prevailing widely used practice was that EPO dose could be increased, decreased, or discontinued every two weeks based only on the most currently available Hb. In addition, individual physicians might also write anemia medication orders. We found by retrospective data analysis that this practice had had an adverse effect on patient Hb (Figure 2).

To enable the new CQI study framework EPO administration was brought under the anemia management team, a core group of caregivers charged with reviewing all patients with a clear understanding of the clinical condition and past response to EPO doses of each patient. Prior to the start, the results of the retrospective analysis and the rationale underlying the new framework were communicated to and discussed with the team by one of the authors (JAL). Orders were to be written only by the anemia management team. Orders by individual physicians were stopped. Senior management endorsed the process to ensure that it was carried out.

Patient-centered EPO administration required first-hand knowledge of each patient and data that might affect the response. Therapeutic decisions were made systematically at one-month intervals by caregivers knowledgeable about each patient's course. These included physicians, nurses and nurse practitioners. Also participating were the unit administrator, nurse manager and physician responsible for implementation of the CQI framework.

To review individual patients, data from HD treatments, medications administered, and laboratory domains were aggregated monthly for the prior 18-months using the aggregate class of reports. Projection on a 140 cm television screen wall mounted in a conference room enabled all team members to see the data simultaneously and participate in decision making. Adjustments to IV iron and EPO were made as needed, consistent with the CQI framework. For acute problems, data aggregated weekly were displayed over a shorter time span. For chronic and perplexing problems, data were aggregated quarterly over several years.

### Controls

The other two other dialysis units administered by The Rogosin Institute served as contemporaneous controls. Both used the EMR in daily practice, were aware of the approach undertaken by the study unit, and of the desirability of reducing EPO administered in view of the upcoming changes in HD treatment reimbursement. An EPO dosing strategy was not imposed, but was left to each unit. Physicians and clinical staff had no training in the processes of CQI.

### Data Analysis

**Figure 3:**
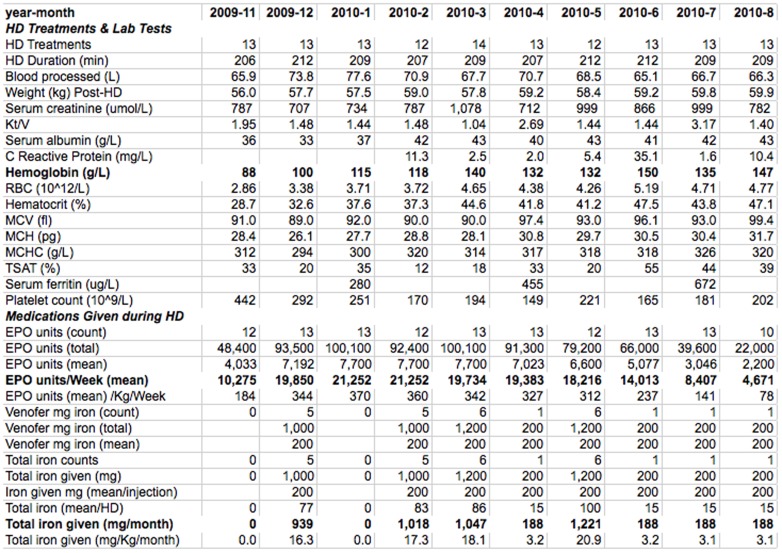
Aggregate report used for CQI in a 44 year old female patient treated by dialysis for 23 years. Although the report was run monthly for the latest 18-months, for clarity only a 10-month time span is shown. This report automatically calculated data for monthly intervals during 2009. Displayed are HD treatments, data relevant to the erythron and iron status, and the EPO and IV iron administered. In November 2009 to January 2010, despite EPO administration of 355–445 units/Kg/week, hemoglobin was low and falling; the low TSAT and relatively low MCH pointed to iron insufficiency. She received 3.4 g of iron intravenously between February and May 2010. An increase in Hb, TSAT, MCH, and serum albumin was evident in May, and continued.

For analysis, the aggregate report was used to abstract data monthly for the historical period 12 months before and the CQI period 18 months after start of the study. Data captured included the number of HD treatments, mean post dialysis weight, Hb, TSAT, serum ferritin, serum albumin, C reactive protein and number of injections, total given, and mean dose of EPO and iron. Mean EPO/week, EPO/Kg/week, IV iron/month, and iron/Kg/month were calculated (Figure 3). Other reports abstracted new and discontinued EPO and IV iron orders. All Hb, TSAT, EPO and iron administration values were exported to JMP software (SAS Institute Inc., Cary, NC) to calculate monthly patient means and percentiles.

Using the Lab - Multiple Patients report, Hb values were abstracted for the two historical 6-month periods before and three 6-month periods after the study start. Data were exported to SAS JMP. Means, standard deviations (SD), coefficients of variation and ranges were calculated as measures of variability, for Hb values of each patient during each 6-month period. Means, medians, SD, ranges, and coefficients of variation were then calculated for all patients.

Mortality per 100 patient years was calculated as is done by USRDS [8], by dividing the number of deaths by the total time in years that the patients were treated by HD, and multiplying by 100.

Medicare Part B allowable charges for EPO and IV iron were calculated using Centers for Medicare and Medicaid Services (CMS) published data (accessed 06/18/2010) and from the amount of each drug given during any month divided by the number of HD treatments during that month. Included were treatments both for patients studied longitudinally and for all others treated in that month.

### Study Approval

The Institutional Review Board did not require informed consent as (1) the study of quality improvement was considered to be a part of routine clinical care of the patients, and (2) the data were analyzed anonymously. The study was approved by the Institutional Review Board of Weill Medical College of Cornell University.

## Results

Mean patient age was 59±16.2 years; 55% were male, 34% White, 46% Black, 20% of other ethnic groups. Primary renal disease was: glomerulonephritis in 35%, hypertension in 30%, diabetic nephropathy in 21%. From February 1, 2010 to July 31, 2011, 17 patients received a kidney transplant, 9 were transferred to peritoneal and nocturnal home HD, 22 to another dialysis unit, and one was lost to follow up.

**Figure 4:**
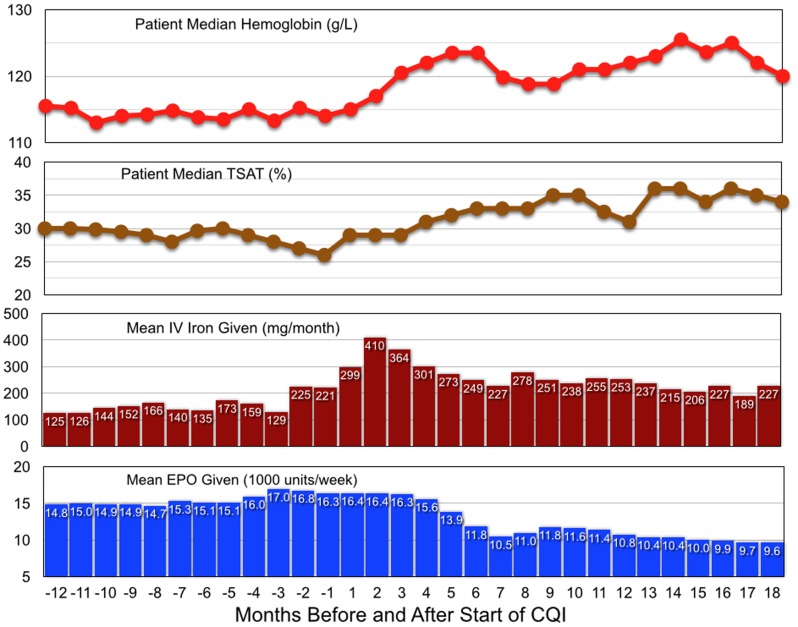
For all study patients, patient median Hb, median TSAT, mean IV iron and mean EPO administered in the protocol period 12 months before study start and the 18-month CQI period.

In the 12-month historical period prior to study start, patient monthly median Hb varied between 113.0 and 115.5 g/L, TSAT between 26% and 30%, with the lowest values immediately before study start (Figure 4). IV iron given varied between 124 mg and 159 mg per month, with larger amounts in the last two months before study start. EPO administration varied between 14,800 and 17,000 units per week.

IV iron administered increased in month 1, peaked at 410 mg/month in month 2, and decreased to ≈250 mg/month by month 6. In months 13–18, an average of 215 mg was given monthly. Hb increased in month 1, and was consistently higher thereafter. In months 13–18 patient monthly median Hb was 120 to 125 g/L. EPO administered decreased slightly in month 4, and further in months 5 and 6. In months 16–18 mean EPO dose was 9,720 units/week.

**Figure 5:**
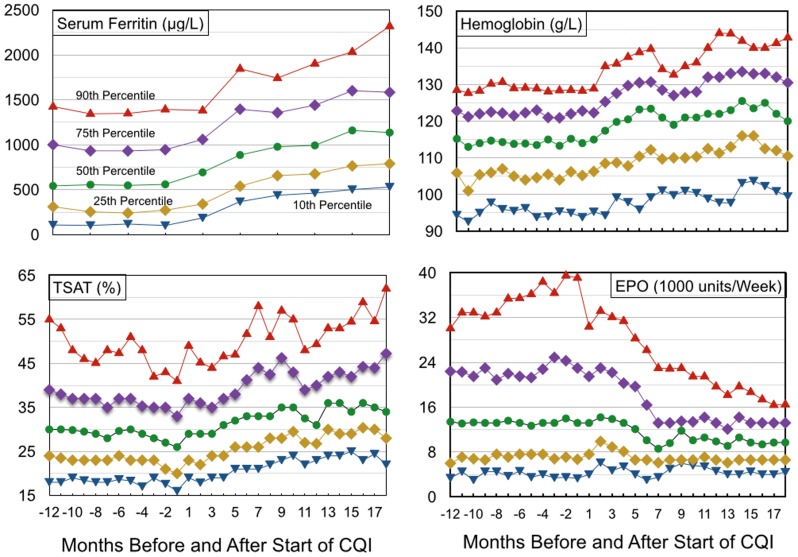
Percentiles of patients by (a) serum ferritin, (b) TSAT, (c) hemoglobin, and (d) administered EPO for 12 months before and 18 months after study start.

To provide more details of changes over time, percentiles of TSAT, serum ferritin, Hb, and EPO administered are displayed monthly. Median serum ferritin, measured 3-monthly, increased from 552 µg/L before study start to 1148 µg/L in months 13–18 (Figure 5a). TSAT levels increased steadily from CQI months 1 to 9 for all percentiles (Figure 5b). In months 13–18, the 10th and 25th percentiles were 23.7% and 29,1% respectively, indicating that iron insufficiency was then uncommon. Hb levels increased steadily from CQI month 1 onward (Figure 5c). In months 13–18, levels were 142.7 g/L for the 90th percentile, 132.5 g/L for the 75th, 122.7 g/L for the 50th, 113.3 g/L for the 25th, and 101.2 g/L for the 10th percentile. EPO administration levels decreased steadily from CQI months 3 to 6, indicating that administered EPO decreased whether initially very high or more modest (Figure 5d). The most striking decreases occurred in the 90th and 75th percentiles. The 50th percentile changed negligibly in months 1–4, and decreased to an average of 9,786 units/week (134 units/Kg/week) in months 13–18.

**Figure 6:**
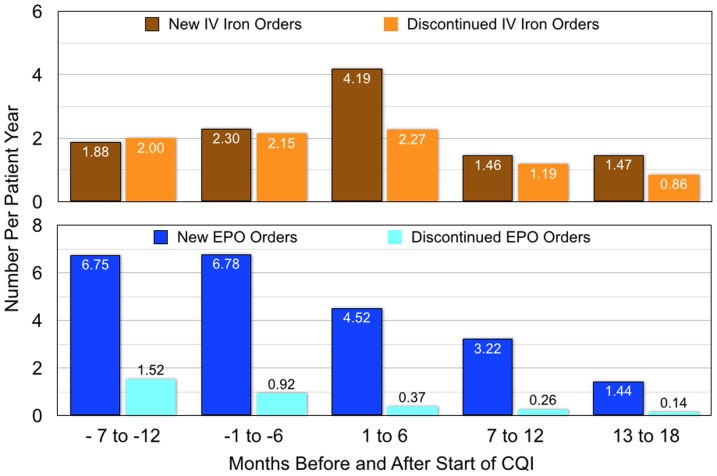
New orders and discontinued orders for IV iron and for EPO for two 6-month periods before and three 6-month periods after the start of the CQI process.

There were 2.10 new and 2.08 discontinued IV iron orders per patient year in the historical period (Figure 6). New IV iron orders doubled in the first 6 CQI months and then decreased to 1.44 per year in months 13–18, when discontinued orders were 0.86 per patient year. New EPO orders, 6.77 per patient year in the historical period, decreased 83% in CQI months 13–18 to 1.12. EPO discontinuation orders, 1.20 per patient year in the historical period, decreased 84% in CQI months 13–18 to 0.19.

With 3 caregivers reviewing the data, 12 caregiver hours were needed at study start for the review of 250 patients; another 3 hours were needed for one caregiver to enter new and changed orders. With experience and increased stability of Hb and TSAT, and of EPO and IV iron orders, fewer caregiver hours were needed: in CQI months 13–18, 6 caregiver hours for the monthly review, and 1.5 caregiver hours to enter new and changed orders. Thus anemia management which consumed 15 caregiver hours per month at study start required only 7.5 caregiver hours per month at the end of the CQI study.

**Figure 7:**
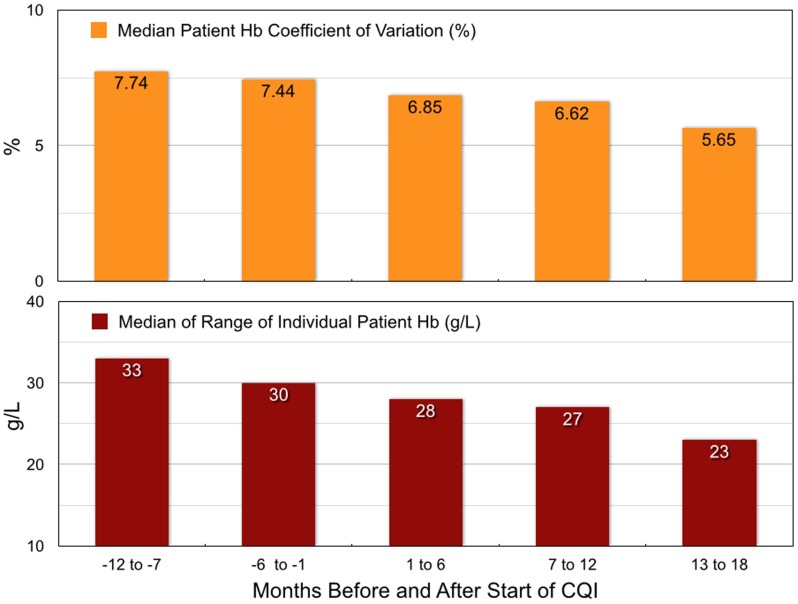
Patient median Hb coefficients of variation and median Hb ranges for two 6-month periods before and three 6-month periods after the start of the CQI process.

The individual patient coefficient of variation and range of Hb values in individual patients were analyzed in five 6-month periods (Figure 7). The coefficient of variation and range decreased after the study start and were least in CQI months 13–18. Coefficients of variation for all Hb measurements for each individual patient, 7.53% (IQR 5.68, 10.63) in the historical period, decreased by 23% to 5.82% (IQR 4.13, 9.04) in CQI months 13–18. The variation of Hb measurements decreased by 27% from 31.5 g/L to 23 g/L.

Using data from all patients treated in the unit from 2004 to 2011 with 10 or more Hb values per year, we analyzed by calendar year the per patient range of Hb levels. This historical perspective showed that from 2004–2009 the range was ≤20 g/L in only 4% of patients, and ≤30 g/L in 15%. In 2011, by contrast, the proportion meeting these low ranges had increased to 9% and 33% respectively.

In the 12 months before CQI start patients were followed for a total of 219.77 years and there were 331 hospital admissions (1.51 per year). During the 18-month CQI period the patients were followed for a total of 297.06 years, and there were 422 admissions to hospital (1.42 per year).

Thirty-five patients died during the CQI study. The mortality rate, 11.78 per 100 patient years, was 42% less than the 2009 United States rate for all dialysis patients of 19.95 per 100 years [8] and did not differ significantly from that before study start. Compared with those who survived the 35 patients who died were 12 years older, had lower serum albumin, lower TSAT, higher C-reactive protein, and a more frequent rate of hospital admission (Table 1). Slightly more EPO was administered in those who died.

**Table 1:** Selected data on patients in the study unit who died and who were alive at the end of the study.

	Died (n = 35)	Alive (n = 215)	p
Age at study start (years)	70.6±14.4	58.6±14.7	<0.001
Admissions per patient year	4.65	1.27	<0.001
Hb (g/L)	112.5 (100.5, 120.5)	121.0 (114.0, 126.0)	<0.001
TSAT (%)	29.5 (23.5, 35.1)	33.0 (29.0, 37.4)	<0.01
Serum ferritin ( µg/L)	1080 (607, 1305)	922 (568, 1328)	NS
Serum albumin (g/L)	37.0 (33.0, 38.5)	40.5 (38.0, 42.0)	<0.001
C-reactive protein (mg/L)	12.3 (6.1, 36.0)	6.33 (2.65, 13.10)	<0.005
EPO given (1000 units/week)	13.53 (7.59, 18.98)	10.25 (6.58, 15.75)	<0.05

Laboratory test results and EPO administered are median values and interquartile range (IQR).

**Figure 8:**
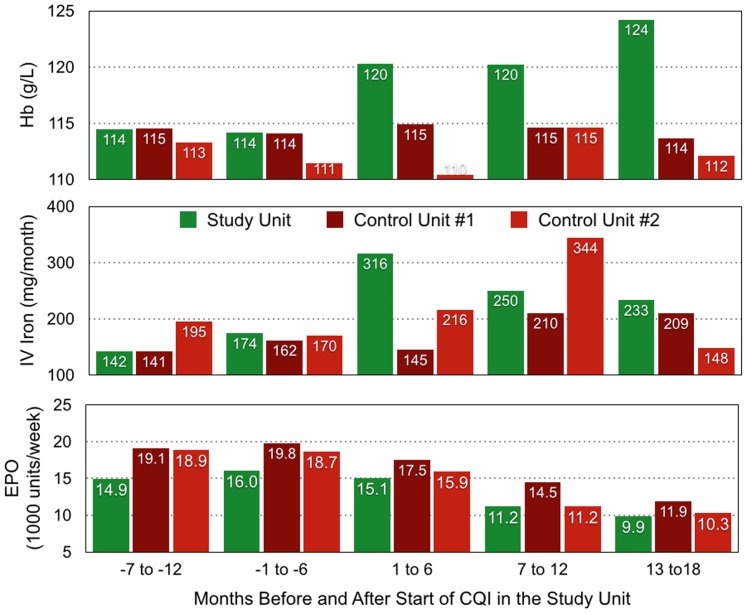
Patient median Hb and mean IV iron and EPO administered in the CQI study and two control units. Data are summarized for two 6-month periods before and three 6-month periods after start of the CQI process in the CQI study unit.

**Figure 9:**
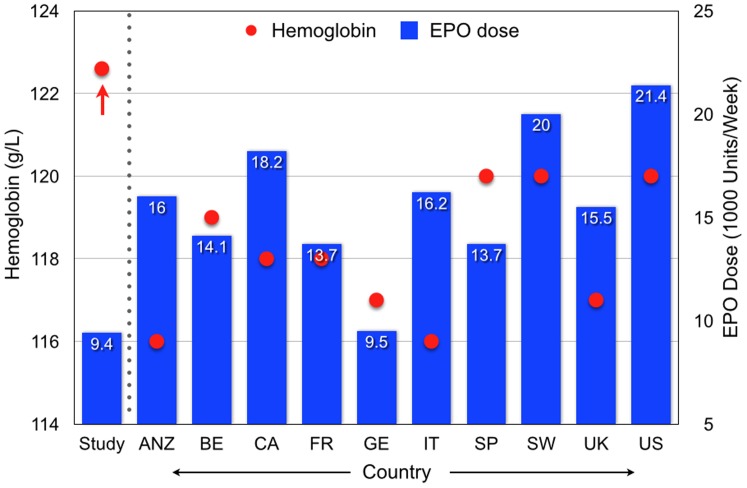
EPO dose and Hb in 2005–2008 in ten countries [26] and in the study unit (Study) in February–July, 2011. (Abbreviations: ANZ =  Australia and New Zealand; BE =  Belgium; CA =  Canada; Fr =  France; GE =  Germany; IT =  Italy; SP =  Spain; SW =  Sweden; UK =  United Kingdom; US =  United States).

In control units #1 and# 2 there were 202 and 175 patients, average age 63.1 and 64.0 years, who received EPO in the 4 months before February 2010. During 216 and 170 years of follow up, deaths were respectively 14.2 and 18.8 per 100 patient years. Little attention was paid to iron administration and EPO was reduced without a defined plan; in unit #2 EPO was reduced in the first 6 months before IV iron administration was increased (Figure 8). TSAT increased to 30.7% and 36.0% by months 13–18 in the two units, serum ferritin to 854 and 1,170 µg/L, but Hb was unchanged (Figure 9). EPO was reduced to a comparable level in all 3 units. New orders for EPO per patient year in months 13–18 were 5.4 and 4.5 times more frequent in the control units than in the study unit; new orders for IV iron were 4.8 and 5.0 times more frequent (Table 2). The median coefficient of variation of Hb was 15% and 34% higher in the control than in the study units.

**Table 2:** Selected data on patients in the study and two control units in months 13–18 after start of CQI in the study unit.

	Study Unit	Control Unit #1	Control Unit #2
Hemoglobin			
Patient median Hb (g/L)	123.6	114	112
Change from 6 months before CQI start in study unit	+8.8%	0%	+1%
Median patient coefficient of variation (%)	5.65%	6.51%	7.55%
EPO			
EPO administered (units/week)	9,867	11,874	10,264
Change from 6 months before CQI start in study unit	−35.5%	−37.7%	−45.5%
EPO new orders per patient year	1.44	7.91	6.44
IV iron			
IV iron administered (mg/month)	233	209	148
IV iron new orders per patient year	1.47	7.04	7.30

The allowable charges for the hematinic medications administered to all patients in the study unit were summarized in five 6-month periods. The allowable charges for EPO administered, $48.99 per HD treatment in the 12-month historical period before CQI start, decreased to $33.69 per treatment in the 13–18 months after CQI start. That of IV iron increased from $4.50 to $7.19.

For comparison, the Medicare 2012 per treatment “bundle” allowance was $56.82 for erythrocyte stimulating agents (EPO and darbepoetin alfa) per HD treatment and $6.54 for IV iron. Thus the allowable charges of $40.88 for CQI study patient use of these two hematinic drugs in months 13–18 was $22.88 (34.8%) less than the Medicare “bundle” allowance.

## Discussion

Standardized protocols are used widely in the United States, where recent EPO use has decreased 40%, with a 6.7% decrease in Hb from 120 to 112 g/L [23]. In the present study the control units used the same EMR as the CQI study unit, but continued a protocol-based approach to anemia treatment. Over 18 study months, EPO administration decreased 41% with little change in Hb. In the CQI study unit, by contrast, EPO administration decreased 42%, but Hb increased by 8.8%; there was also less Hb variation than in the controls (Table 2). EPO administered weekly in the CQI study unit was also much less than that in 9 countries in a recent study of a randomly selected group of 300 patients from each of 10 countries [24]. Yet, Hb in the CQI unit was 2.6 to 6.6 g/L higher than in each of these 10 countries.

There have been concerns about adverse effects of Hb>120 g/L in renal failure [16]–[18]. Re-analysis, however, showed the lowest mortality was associated with the highest achieved Hb [25], [26]. In other studies, patient mortality was highest with Hb≤110 g/L, lowest with Hb>120 g/L [27]–[31]. In the CQI study unit, patient median Hb was 121.0 g/L in survivors, 112.5 g/L in those who died. Hospital admissions were 3.66 times more frequent in those who died. Arranged in ranks (≤100; 100.1–110; 110.1–120; >120 g/L) Hb distribution differed in the two groups (χ^2^_[3]_ = 19.6, p<0.0003). These data are consistent with an association of mortality with age and morbidity rather than with high Hb.

We investigated the importance of iron and effect of Hb on survival in a 9.5-year retrospective study of 1774 patients [19]. Multifactorial analysis demonstrated the favorable effect of Hb>120 g/L. As compared with the 442 patients with Hb>120 g/L, the hazard ratios for death for Hb 110–120, 100–110, and <100 g/L were respectively 1.67, 2.69, and 2.83. In the last 3 months of the present study, TSAT was ≤20% in 5%, ≤25% in 11% of patients in the CQI study unit, (Table 3), while in control units #1 and #2 respectively the proportions were higher: ≤20% in 17% and 11% of patients, ≤25% in 40% and 27%.

**Table 3:** Percentage of patients with various TSAT levels in the last 3 months of three studies over 14 years from 1997 to 2011.

IV iron administration	Years of Study	CQI	TSAT (%)			
			≤15	≤20	≤25	≤30
Previous Studies						
Iron insufficient at study start, prospective [12]	1996–1997	Yes	1	9	30	48
Clinical practice, retrospective [17]	1998–2007	No	5	16	29	51
Present Study						
CQI Study Unit	2009–2011	Yes	1	5	11	26
Control Unit #1	2009–2011	No	5	17	40	62
Control Unit #2	2009–2011	No	5	11	27	45

This suggested that the conventional method of IV iron administration in intermittent courses, without systematic feedback, was associated with suboptimal results; patients were probably cycling between iron deficiency and repletion, without being continuously iron sufficient. In the CQI study group only 6% of patients had TSAT ≤20% in the last 3 months, suggesting that continuous long-term modulation of interrelationships between IV iron, EPO dose and Hb was probably enabled by the CQI process.

In contrast to the current widely used approach that strongly encourages application of population-based guidelines or protocols to individual patient care, CQI enables clinicians to assess variables such as severity of symptoms, illness, comorbidity, and other clinical nuances [32] with clinically pertinent reports immediately available at the point-of-care. Skilled clinicians can thereby generate patient and practice specific knowledge on a day-to-day basis for each patient as an individual, and practice medicine based on scientific judgment conditioned by the totality of available up-to-date evidence of the state of the patient [33].

The application to patient care of protocols without critical oversight of details of individual patients neither promotes nor enables consideration by caregivers of details of the patient illness; it may result in treating patients without regard specifically to factors such as age, race, gender, and co-morbid conditions that may impair or enhance treatment responses. This is particularly so with protocols based on ensuring that certain predetermined numbers are met. By contrast, the CQI process promotes and enables attention to many details of care of patients as individuals, and as individual members of a patient population. It enables caregivers to inquire of the reasons why therapeutic objectives were not met, promotes correction of underlying problems or complications, and does so repeatedly and particularly.

We have shown that a disciplined, classic CQI process wedded to an appropriately designed EMR can lead to better outcome and reduce cost. Neither alone suffices. To facilitate decisions repeatedly over long time spans access serially to relevant time-oriented information including laboratory tests and medications is essential; these data must be organized to display feedback over time between treatments and their effects. Without an EMR that so displays data in spreadsheet (or graphic) metaphor the task is personnel intensive and overwhelming. Needed for introduction and implementation of CQI is clinical and administrative leadership and education and encouragement of physicians, nurses, and other caregivers.

The present study has limitations. It was applied to a single clinical problem, and needs testing with others. It was tested in a dialysis unit treating patients with chronic renal failure, and requires testing in other populations and practice environments. It should also be tested with other EMRs that display feedback over time between treatments and their effects.

## Conclusions

Medical therapies are often based on randomized studies where patient variability is intentionally filtered out, or protocols assembled from relevant literature; these are rarely tested in diverse populations. Physicians are also bereft of tools and techniques to apply randomized study results or protocols to each particular individual patient. CQI is an iterative process to understand and contain the effects of variability that, in clinical medicine, stems from the biologic uniqueness of individual patients and their co-morbid conditions.

A recent Institute of Medicine report concluded: “Although unprecedented levels of information are available, patients and clinicians often lack access to guidance that is relevant, timely, and useful for the circumstances at hand. Overcoming this challenge will require applying computing capabilities and analytic approaches to develop real-time insights from routine patient care, disseminating knowledge using new technological tools, and addressing the regulatory challenges that can inhibit progress” [34].

The CQI process is sustainable, practical and appropriate for most complex, expensive therapies now used in chronic disease patient care. We suggest that it provides a model to address both the issues raised by the recent Institute of Medicine report, and an approach to design of methodologies to optimize physician decision making for individual patient care in heterogeneous patient populations.
